# Synthesis, Characterization and In Vitro Antibacterial Evaluation of *Pyrenacantha grandiflora* Conjugated Silver Nanoparticles

**DOI:** 10.3390/nano11061568

**Published:** 2021-06-15

**Authors:** Arinao Murei, Karen Pillay, Patrick Govender, Ntevheleni Thovhogi, Wilson M. Gitari, Amidou Samie

**Affiliations:** 1 School of Mathematics and Natural Science, Microbiology Department, University of Venda, Private Bag x5050, Thohoyandou 0950, South Africa; mureiarinao@gmail.com; 2 Department of Biochemistry, University of Kwa-Zulu-Natal, Private Bag X54001, Durban 4001, South Africa; muthusamy@ukzn.ac.za (K.P.); govenderpt@ukzn.ac.za (P.G.); 3 Biomedical Research and Innovation Platform (BRIP), South African Medical Research Council, P.O. Box 19070, Tygerberg, Cape Town 7500, South Africa; ntevhe.thovhogi@mrc.ac.za; 4 Environmental Remediation and Water Pollution Chemistry Group (ERWPCG), Department of Ecology and Resource Management, School of Environmental Sciences, University of Venda, Private Bag X5050, Thohoyandou 0950, South Africa; mugera.gitari@univen.ac.za

**Keywords:** antibacterial activity, silver nanoparticles, conjugates, transmission electron microscopy, medicinal plants, agar diffusion assay, MIC, MBC, FICI, synergy

## Abstract

In the present study, silver nanoparticles (AgNPs) were synthesized using both the chemical and biological methods and conjugated with *Pyrenacantha grandiflora* extracts. These were then characterized and evaluated for antimicrobial activities against multi-drug resistant pathogens, such as methicillin-resistant *Staphylococcus aureus* (MRSA), *Klebsiella pneumonia,* and *Escherichia coli*. Nanoparticles were analyzed with UV-visible spectrophotometer, transmission electron microscopy (TEM), and energy dispersive X-ray analysis (EDX). Silver nanoparticles, *P. grandiflora* extracts, and the conjugates were also analyzed with Fourier transform infrared spectroscopy (FTIR). As a result, quasi-sphere-shaped AgNPs with sizes ranging from 5 to 33 nm and spherically shaped AgNPs with sizes ranging from 3 to 25 nm were formed from chemical and biological synthesis, respectively. A well diffusion assay showed that the activity of silver nanoparticles was most improved with acetone extract against all tested bacteria with diameters in the range of 19–24 mm. The lowest MIC value of 0.0063 mg/mL against MRSA was observed when biologically synthesized AgNPs were conjugated with acetone and water extracts. Chemically synthesized silver nanoparticles showed the lowest MIC value of 0.0063 mg/mL against *E. coli* when conjugated with acetone and methanol extracts. This study indicates that silver nanoparticles conjugated with *P. gandiflora* tubers extracts exhibit strong antibacterial activities against multi-drug resistant bacterial pathogens. Therefore, biosynthesized conjugates could be utilized as antimicrobial agents for effective disease management due to the synergistic antibacterial activity that was observed.

## 1. Introduction

There is an urgent need for novel antibacterial agents. Therefore, research has focused on developing several strategies to address this issue. This has been done by trials in making novel antibacterial agents or artificially adjusting the action of existing/momentarily effective antibiotics [1]. In the present scenario, medicinal plants and silver nanoparticles are emerging to address the challenge due to their efficacy as antimicrobial agents. A number of studies have proved that silver nanoparticles possess antimicrobial, anti-inflammatory, anti-angiogenic, anticancer, and antiviral properties with several advantages, such as less toxicity, enhanced biodegradability, and bioavailability even in industrial applications [2,3,4,5].

Plants and their derived products have long been used by humans for medicinal purposes. It is estimated today that about 80% of the world’s population uses botanical preparations as medicines to cover their health needs [6]. Thus, there are about half a million therapeutic plants, far and wide; and for the vast majority of them, their medicinal activity has not yet been explored, although their restorative activities could be unequivocal in the treatment of present and future diseases [7]. Several studies have been done in South Africa to highlight antibacterial activities of some medicinal plants. For example, *Pyrenacantha grandiflora* Baill was shown to have antibacterial activity [8].

To develop a novel treatment, two or more effective treatment strategies can be combined. This study is based on the assumption that the co-existence of two or more different molecules with entirely different modes of action in the form of a hybrid molecule will produce a synergistic effect. Hybrid molecules thus could offer advantages, such as dosage compliance, minimizing toxicity, and overcoming drug resistance when compared to the parent counterparts [9]. In most studies, an increase in antimicrobial activity was reported after conjugation of the antibiotic with the nanoparticles. In 2014, silver nanoparticles (AgNPs) were reported to improve the antimicrobial activity of ampicillin [10]. Silver nanoparticles were also reported to have antibacterial activity when conjugated with plant extracts [11]. We have previously studied the antimicrobial activity of *P. grandiflora* extracts in association with silver nanoparticles and antibiotics [12]. The present study focused on the evaluation of the antibacterial activity of *Pyrenacantha grandiflora* Baill extracts when conjugated with silver nanoparticles against problematic pathogenic bacteria (*Staphylococcus aureus* (MRSA), *Escherichia coli, Klebsiella pneumoniae*).

## 2. Materials and Methods

### 2.1. Microorganism and Growth Conditions

The microorganisms that were used in this study include *Magnetospirillum magnetotacticum* MS-1 (DSMZ, Braunschweig, Germany) which was grown in a modified chemically defined growth medium supplemented with Isogro (Merck, Modderfontein, South Africa), methicillin-resistant *Staphylococcus aureus* (ATCC 25,923), and methicillin-susceptible *Staphylococcus aureus* (ATCC 33,594) sub-cultured on mannitol salt agar, *Escherichia coli* (ATCC 35,218 and 25,922) sub-cultured on MacConkey agar, and *Klebsiella pneumonia* (ATCC 700,603) sub-cultured on nutrient agar (Rochelle, SA). An inoculum of each bacterial strain was suspended in 5 mL of Mueller Hinton broth (Rochelle, Johannesburg, South Africa) and incubated for 3 h at 37 °C. The cultures were diluted with Mueller Hinton broth and adjusted to give a concentration of bacterial cells equivalent to a 0.5 McFarland standard prior to the antibacterial testing.

### 2.2. Chemical Synthesis of Silver Nanoparticles

#### 2.2.1. Silver Nanoparticles Preparation

Silver nanoparticles were prepared using the chemical reduction method, as per the Turkevich protocol [13]. Briefly, 1 mM of silver nitrate (AgNO_3_) powder (Sigma, St. Louis, MI, USA) and 1% tri-sodium citrate of analytical grade purity and were allowed to react. A total of 100 mL of 1 mM AgNO_3_ was heated to boiling to which 5 mL of 1% tri-sodium citrate was added drop by drop. During the process, the solution was mixed vigorously. The solution was heated until a color change was evident (yellowish-brown). The reaction solution was removed from the heating plate and stirred until cooled to room temperature.

#### 2.2.2. UV-Vis Spectrum Analysis

The characterization of silver nanoparticles was done using a UV-Visible spectrophotometer (Specord 210, Analytikjena spectrometer, Mettler Toledo, Mumbai, India). The reduction of silver nitrate to silver nanoparticles by sodium citrate was confirmed by observing a broad absorbance peak between 400–500 nm.

#### 2.2.3. High Resolution-Transmission Electron Microscopy

Further characterization was done using high-resolution transmission electron microscopy (HR-TEM) studies. The sample was prepared by placing a drop of the nanoparticle solution onto a carbon-coated copper transmission electron microscope (TEM) grid. The sample was then dried under an infrared lamp for a period of 45 min for the solvent to evaporate. High-resolution TEM images were obtained on JEOL transmission electron microscope, model no 2100 (Nottinghamshire, United Kingdom) operated at an accelerating voltage of 200 kV and 0.23 nm resolution.

### 2.3. Biological Synthesis of Silver Nanoparticles

#### 2.3.1. Cultivation of Magnetospirillum Magnetotacticum Bacteria

Silver nanoparticles were synthesized using *Magnetospirillum magnetotacticum* MS-1. These bacteria were cultured in chemically defined growth media prepared with slight modification. Isogro was added to the media to enhance the growth of bacteria by shortening the lag phase of growth, thereby resulting in high bacterial yield. Silver nitrate was used as the source of metal in the growth media. The MS-1 cells were grown for 4 days at 30 °C in airtight 50 mL falcon tubes wrapped with parafilm because they grow well in micro-aerobic conditions. Tubes were covered with foil to prevent photodegradation of silver nitrate in the media. The synthesis of silver nanoparticles was initially confirmed by a color change in the media.

#### 2.3.2. Analysis of Silver Nanoparticles Synthesis by HR -TEM

A high-resolution transmission electron microscopy (HR-TEM, Thermo Fisher Scientific, New York, NY, USA)) was used to detect whether silver nanoparticles were produced by *Magnetospirillum magnetotacticum* cells. The sample was prepared by placing a drop of the bacterial culture onto a carbon-coated copper TEM grid. The sample was then dried under an infrared lamp for a period of 45 min. High-resolution TEM images were obtained on a JEOL TEM, Model No. 2100, operated at an accelerating voltage of 200 kV and 0.23 nm resolution.

#### 2.3.3. Isolation of Silver Nanoparticles

The isolation of silver nanoparticles from *Magnetospirillum magnetotacticum* culture was performed using MACS magnetic separation column (Miltenyl Biotec, Magdeburg, Germany). Briefly, *M. magnetotacticum* bacterial cells were suspended in 20 mM HEPES-4 mM EDTA (pH 7.4) and broken open by sonication for 5 min at 21 °C. The unbroken cells were removed by centrifugation at 9000 rpm for 30 min. The supernatant was harvested and passed through the MACS magnetic separation column, following the manufacturer’s protocol. Unbound magnetic particles were washed using 10 mM HEPES-200 mM NaCl (pH 7.4), and thereafter the silver nanoparticles were eluted with 10 mM HEPES (pH 7.4).

### 2.4. Preparation of Plant Extracts and Characterization

*Pyrenacantha grandiflora* tubers were collected in cool, dry conditions. The harvested tubers were washed with distilled water to remove any contaminants. They were subsequently cut into smaller pieces and placed in a drying room for four weeks. The bioactive compounds of *P. grandiflora* were extracted using methanol, hot water (distilled water), and acetone. For hot water extract, the stock solution was prepared by adding 100 g of tubers powder in 1 L of distilled water, then boiled for 15 min and followed by cooling and kept at 4 °C. For methanol and acetone extraction, 100 g of dried tuber were added into 1 L of each solvent and allowed to homogenize for 24 h at room temperature. All mixtures were filtered through Whatman filter papers, and proper actions were taken to ensure that potential active constituents are not lost, distorted, or destroyed during the preparation of the extracts from plant samples. Filtrates were concentrated using rotary evaporators (Rota vapor-R, Buchi, Switzerland). Different temperatures were used to evaporate extract solvents: acetone at 50 °C and methanol at 60 °C. Hot water extract was concentrated using the freeze dryer. All concentrated samples were further dried into powder at room temperature. A FTIR spectrophotometer was used to detect various functional groups responsible for biological activities from the crude extract of *P. grandiflora* tubers.

### 2.5. Preparation of Plant Extracts Conjugated with Silver Nanoparticles and Characterization

Non-covalent modification of plant extracts with silver nanoparticles was applied for the generation of hybrid molecules [14]. Synthesized silver nanoparticles from both chemical and biological methods were mixed with 10 mg/mL of each plant extracts (acetone, water, and methanol extracts) and incubated at 4 °C for 24 h.

All conjugates were analyzed using Fourier transmission electron microscopy (FTIR) in a range of 400–4000 cm^−1^ to detect various functional groups formed after conjugation that are responsible for biological activities. A precise volume of 500 µL of the conjugates was then placed on the sample chamber of FTIR spectrophotometer and the spectra were recorded in the scan range of 400–4000 cm^−1^ with a resolution of 4 cm^−1^ in a Nicolet Avatar 330 FTIR spectrometer.

### 2.6. Antimicrobial Activities of the Nanoparticles and Conjugates

#### 2.6.1. Well Diffusion Assay

The antibacterial activities of conjugates were initially determined by the well diffusion method. The zones of inhibition were recorded in millimeters (mm). Briefly, bacterial suspensions were prepared with a turbidity of 0.5 McFarland Standard. Mueller-Hinton agar plates were inoculated with *E. coli*, *K. pneumonia*, and *S. aureus*. Wells with a diameter of 6 mm were cut using a cork borer and filled with 30 μL of the conjugates or reference samples (plant extract and silver nanoparticles). Distilled water was used as a negative control. Plates were incubated for 24 h at 37 °C. After incubation, the growth inhibition zone diameters were measured.

#### 2.6.2. Microdilution Assay

The minimum dilution of all extracts plus conjugate samples that inhibit the growth of the microorganisms were denoted as minimum inhibitory concentration (MIC) [15]. Distilled water was used as the negative control and gentamycin as a positive control. After adding INT, the results were read by observing the color change and determining the MIC. All the conjugates that show activity (reduced or no color change) were inoculated again in the agar plate and incubated overnight to determine the minimum bactericidal concentration (MBC).

#### 2.6.3. Fractional Inhibition Concentration Index (FICI) Calculations

Determination of the mutual influence of *P. grandiflora* tuber extracts and silver nanoparticles in conjugates was done using fractional inhibition concentration index by the following formula:

$$FICI=\frac{MIC\;of\;AB}{MIC\;of\;A}+\frac{MIC\;of\;AB}{MIC\;of\;B}$$

where *AB* represents a combination of *P. grandiflora* tubers extracts (A) and silver nanoparticles (B). Results were interpreted as synergy (FICI ≤ 0.5), antagonism (FICI > 4) and no interaction or additive (FICI > 0.5–4.0).

## 3. Results

### 3.1. Analysis of Silver Nanoparticles

Silver nanoparticles were successfully synthesized using the chemical method. The color of silver nitrate solution changed from colorless to a yellow color when 1% of sodium citrate is added. This color originates from coherent electron motion in the colloidal solution, giving rise to a characteristic absorption of light at a wavelength of 400–500 nm to confirm the synthesis of nanoparticles. The presence of silver nanoparticles was further confirmed by ultra-violet visible spectroscopic studies showing a peak at 434 nm (maximum absorbance).

### 3.2. Transmission Electron Microscopy Analysis of Silver Nanoparticles

**Figure 1:**
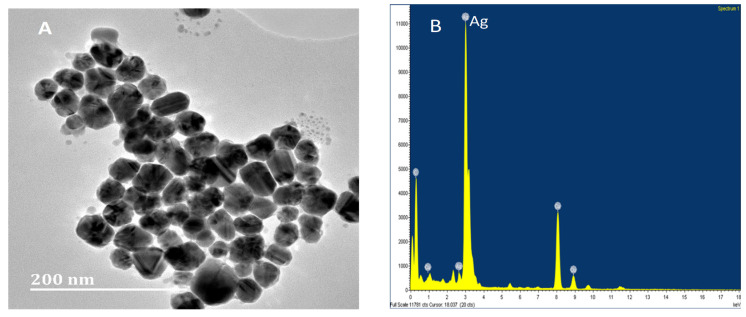
Analysis of chemical synthesis of silver nanoparticles. Images showing the TEM picture of silver nanoparticles (**A**) and EDX pattern (**B**).

The morphology of nanoparticles refers to the quasi-spheres shape. In the higher magnification image, silver nanoparticles of 5–33 nm size were also observed (Figure 1A). However, the average size of the nanoparticles was found to be 13 nm. The EDX analysis confirmed that the particles were composed of elemental silver (Figure 1B).

### 3.3. TEM Analysis of Biologically Synthesized Silver Nanoparticles

**Figure 2:**
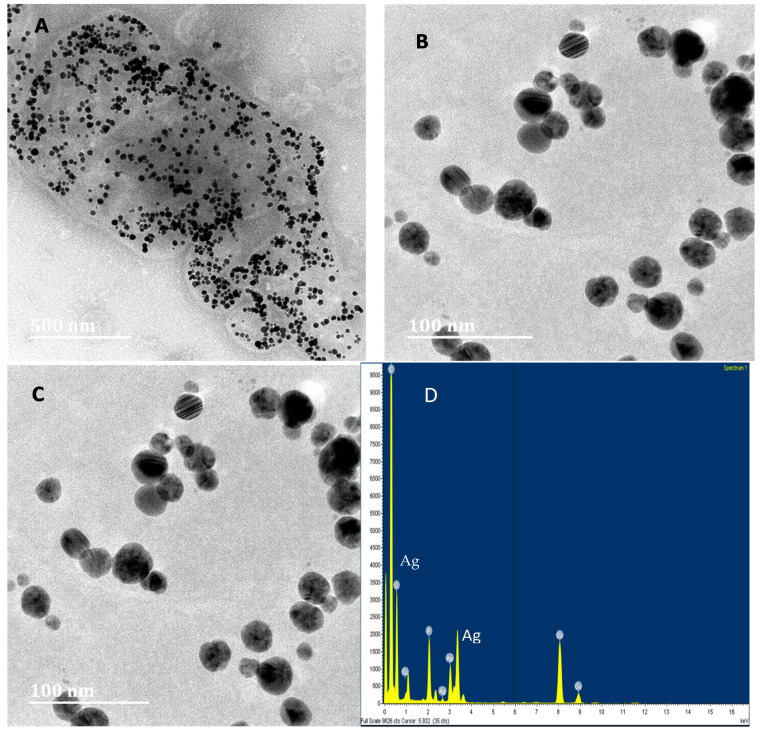
Analysis of biological synthesis of silver nanoparticles. TEM images of *Magnetospirillum magnetotacticum* with silver nanoparticles (**A**), silver nanoparticles (**B**), lattice fringes (**C**) and EDX pattern (**D**).

The synthesis of nanoparticles by a microorganism was initially confirmed by the change of color in the media [16]. In this study, the color of modified chemically defined media with MS-1 cells changed from orange to deep brown. HR-TEM was used to evaluate the cell morphology of *Magnetospirillum magnetotacticum* (Figure 2A) and also revealed that silver nanoparticles were present within the cells (Figure 2B). Synthesized silver nanoparticles were spherical shaped, and their size ranged from 3 to 25 nm. The selected diffraction pattern (Figure 2C) indicated that these silver nanoparticles are formed through the reduction of metal ions. The EDX analysis confirmed that the particles were composed of elemental silver (Figure 2D).

### 3.4. Plant Extracts Analysis

**Figure 3:**
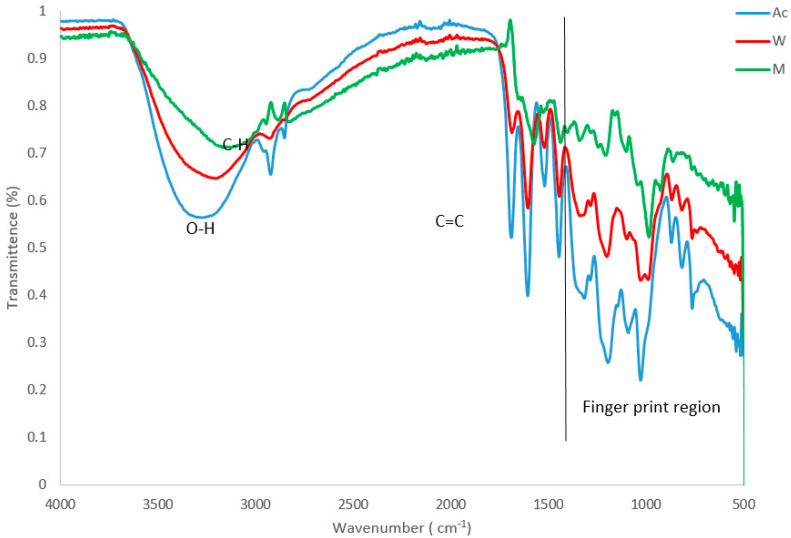
FTIR spectrum of *P. grandiflora* tubers extract prepared using different solvent where: Ac = acetone extract, W = water extract, and M = methanol extract.

The FTIR spectroscopic studies in the extracts and conjugates revealed different characteristic peak values with various functional compounds in the extracts. Important absorption frequencies that appeared in the functional group region, as well as the fingerprint region of the spectra, were noted. The FTIR spectrum of *P. grandiflora* tuber extracts (prepared in different solvents) are given in Figure 3. The data on the peak values and the probable functional groups were obtained by FTIR analysis. This analysis revealed the presence of a hydroxyl group (OH), C-H stretching and C=C carboxyl ranging from 3306 to 3153, 2923–2894, and 1689–1557, respectively. Hence, these functional groups observed in different *P. grandiflora* extracts probably indicate the presence of various phytochemicals and other medicinally important compounds.

### 3.5. Analysis of Silver Nanoparticles Conjugated with Plant Extracts

**Figure 4:**
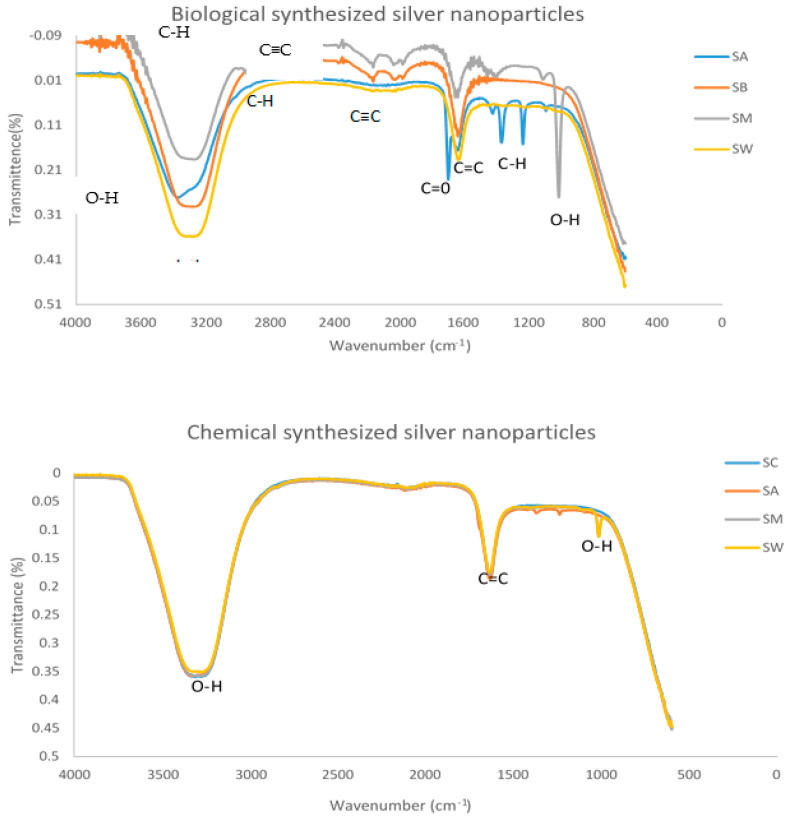
FTIR spectrum of plant extracts conjugated with silver nanoparticles. SB (Biologically synthesized silver nanoparticles), SA (Silver nanoparticles and acetone extract), SM (Silver nanoparticles and methanol extract), SW (Silver nanoparticles and water extract) and SC (Chemically synthesized silver nanoparticles).

Six conjugates were made from silver nanoparticles and plant extracts, three from chemically synthesized silver nanoparticles and three from biologically synthesized silver nanoparticles. Conjugates were silver nanoparticles-methanol extract (SM), silver nanoparticles-water extract (SW), and silver nanoparticles-acetone extract (SA). The measurements of FTIR were carried out in order to recognize the existence of different functional groups that results after conjugation, as shown in Figure 4. Most conjugated samples compared to reference samples showed the formation of -OH carbonyl and carbonyl (C=C) group, however, C-H group, (C=O) group, and C≡C group was also observed.

### 3.6. Well Diffusion Assay

**Figure 5:**
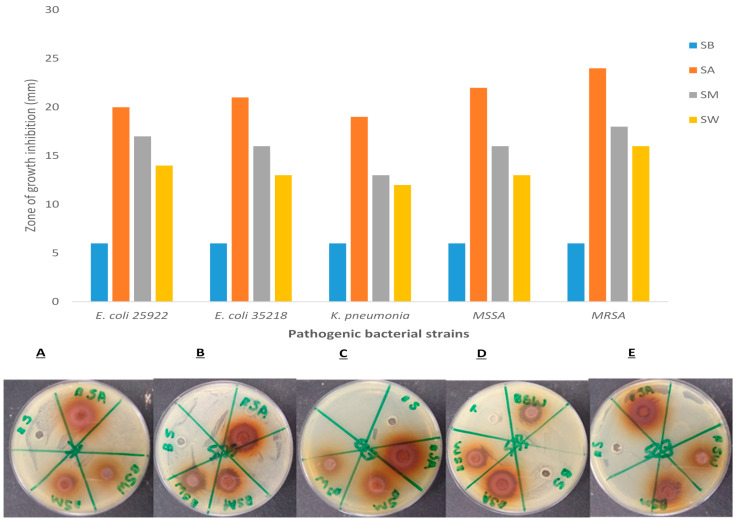
Antibacterial activity of P. grandiflora tuber extracts conjugated with biologically synthesized silver nanoparticles against selected bacterial strains; E. coli 25,922 (**A**), E. coli 35,218 (**B**), K. pneumonia (**C**), Methicillin-susceptible Staphylococcus aureus (MSSA) (**D**), Methicillin-resistant Staphylococcus aureus (MRSA) (**E**). where SB (Biologically synthesized silver nanoparticles), SA (Silver nanoparticles and acetone extract), SM (Silver nanoparticles and methanol extract), SW (Silver nanoparticles and water extract).

Well diffusion assay was used to determine the antimicrobial activity of *P. grandiflora* tuber extracts when conjugated with silver nanoparticles. Biologically synthesized nanoparticles showed the smallest zone of growth inhibition of 6 mm in diameter in all bacteria tested in this study. However, a higher zone of growth inhibition of 24 mm was observed with biological synthesized silver nanoparticles conjugated acetone extract against methicillin-resistant *Staphylococcus aureus* (MRSA) (Figure 5). In all bacterial strains tested with biologically synthesized silver nanoparticles, silver nanoparticles conjugated acetone extract exhibited the highest antibacterial activity with the diameter of inhibition ranging from 19 to 24 mm, followed by silver nanoparticles conjugated with methanol extract with a diameter ranging from 13 to 18 mm. Silver nanoparticles conjugated water extract showed less efficacy of antibacterial activity when compared to the other conjugates.

**Figure 6:**
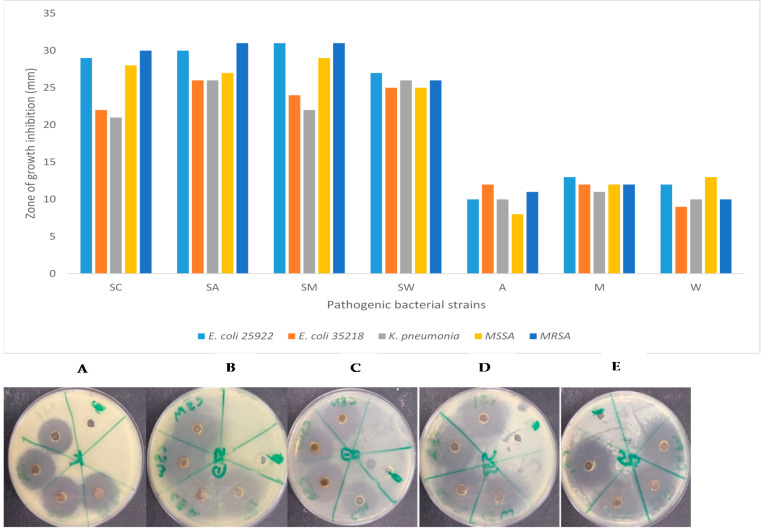
Antibacterial activity of *P. grandiflora* tuber extracts conjugated with chemically synthesized silver nanoparticles against selected bacterial strains; E. coli 25,922 (**A**), E. coli 35,218 (**B**), K. pneumonia (**C**), MSSA (**D**), MRSA (**E**). SC (Chemically synthesized silver nanoparticles), SA (Silver nanoparticles and acetone extract), SM (Silver nanoparticles and methanol extract), SW (Silver nanoparticles and water extract).

Chemically synthesized silver nanoparticles exhibit good antibacterial activity against all tested bacterial strains with the zone of inhibition diameter ranging from 21 to 30 mm (Figure 6). The highest antibacterial activity (31 mm) was exhibited by water extract conjugated silver nanoparticles against *E. coli* 25,922 and methicillin-resistant *Staphylococcus aureus* (MRSA) and also by acetone extract conjugated silver nanoparticles against *S. aureus*. Water extract conjugated with silver nanoparticles also exhibited good antibacterial activity with a zone inhibition ranging from 25 to 27 mm.

### 3.7. Microdilution Assay

Antimicrobial activity has been reported as noteworthy for plant extracts when the MIC values were <1.00 mg/mL. In this study, the concentration used ranged from 0.8 to 0.0063 mg/mL of plant extracts and their conjugates. All plant extracts showed very good antibacterial activity when conjugated with silver nanoparticles (Table 1). The lowest MIC value of 0.0063 mg/mL was observed when biologically synthesized silver nanoparticles are conjugated with acetone and water extracts and tested against methicillin-resistant *Staphylococcus aureus* (MRSA). Chemically synthesized silver nanoparticles also showed the lowest MIC value of 0.0063 mg/mL against *E. coli* 25,922, and when conjugated with acetone and methanol extracts, similar activity was observed against both *E. coli* and *K. pneumonia,* respectively.

### 3.8. Minimum Bacterial Concentration (MBC)

Only a few conjugates were able to kill the tested bacterial strains. Methicillin-susceptible *Staphylococcus aureus* (MSSA) was killed by all conjugates of chemically synthesized silver nanoparticles (Table 2). However, *E. coli* 25,922 was killed by unconjugated silver nanoparticles and silver nanoparticles conjugated to acetone extract with MBC values of 0.4 and 0.05 mg/mL, respectively, whereas *E. coli* 25,922 was killed by silver nanoparticles conjugates to methanol extract with an MBC value of 0.0063 mg/mL. None of the biologically synthesized silver nanoparticle conjugates were able to exhibit minimum bactericidal concentration.

### 3.9. Fractional Inhibition Concentration Index (FICI) calculations

Fractional Inhibition Concentration Index was calculated for the results obtained from MIC, and the values are shown in Table 3. A total of six samples from biologically and chemically synthesized silver nanoparticles conjugated plant extracts were tested against five bacterial pathogens. A total of 7 synergies (23.3%) were observed, 9 (30%) were additive and 14 (46%) were antagonism.

## 4. Discussion

Silver nanoparticles have been reported to have a wide range of applications; they are known for their antimicrobial properties and has been used for years in the medical field for antimicrobial applications and even been shown to prevent HIV binding to host cells [17]. In recent years, many researchers have focused on the development of modified or novel synthetic strategies for silver nanoparticles, in contrast to the use of conventional methods which are strongly associated with toxic environmental footprints [18,19]. This study reports on the antimicrobial activities of *P. grandiflora* tuber extracts when conjugated with chemically and biologically synthesized silver nanoparticles against pathogenic bacteria.

Various microbes are known to reduce the Ag^+^ ions to form silver nanoparticles, most of which are found to be spherical particles [20]. In biological synthesis of silver nanoparticles, *Magnetospirillum magnetotacticum* bacteria were used, and they produced spherical silver nanoparticles with the size ranging from 3 to 25 nm. These results are similar to those obtained by Abhilash et al. [21]. Studies have found that many microorganisms can produce inorganic nanoparticles through intracellular or extracellular routes [16], and in this study, TEM imaging of *M. Magnetotacticum* revealed that silver nanoparticles were produced intracellularly.

Other than the *Magnetospirillum* species of bacteria, Klaus and coworkers have shown that the bacterium *Pseudomonas stutzeri* AG259 (isolated from a silver mine) when placed in a concentrated aqueous solution of silver nitrate, played a major role in the reduction of the Ag^+^ ions and the formation of silver nanoparticles of well-defined size [22]. However, *Magnetospirillum* used in this study are mesophilic facultative anaerobic that survive at a very low concentration of oxygen [23]. Hence, the level of oxygen was kept at a minimum by tightly wrapping culture tubes with a parafilm. On the other hand, silver nanoparticles were also chemically synthesized according to the protocol provided by Turkevich [13] and characterization was done by UV-Vis spectroscopy and TEM.

Synthesized silver nanoparticles from biological and chemical methods were conjugated to plant extracts from *P. grandiflora* tuber. Most studies report reduction and stabilization of silver ions by the combination of biomolecules, such as proteins, amino acids, enzymes, polysaccharides, alkaloids, tannins, phenolics, saponins, terpenoids, and vitamins, which are already established in the plant extracts having medicinal value [16]. Moreover, plants have been reported to facilitate silver nanoparticle syntheses [16]. Different parts of the plants, which include barks, roots, and leaves, have been used to reduce silver nitrates into nanoparticles. With increasing intensity of extract during the period of incubation, another research study on silver nanoparticles showed a gradual change in color of the extracts to yellowish-brown with callus extract of the salt marsh plant, *Sesuvium portulacastrum* [24].

In the present work, FTIR spectral analysis of the tubers of *P. grandiflora* showed the presence of phytochemicals carrying a hydrogen-bonded –OH functional group. It is well-established that the hydroxyl functional group is an integral part of most of the phenolic phytochemicals, such as flavonoids and tannins [25]. The phytochemical screening of tubers of *P. grandiflora* certainly encourages future advanced research activities on chromatographic isolation of these compounds in their pure state using either high performance liquid chromatography (HPLC), column chromatography or gas chromatography and furthermore, to evaluate in detail the in vivo biological activities of such isolated compounds.

In order to investigate bond formation between the functional groups present on the *P. grandiflora* tubers extracts and silver nanoparticles, it was necessary to analyze the infra-red (FT-IR) spectrum of the samples. This indicates whether the plant extract has bound with silver nanoparticles and between which functional groups this bond has formed. Previous studies have indicated that neither the keto nor the carboxyl groups present on the antibiotic molecule are involved in binding to the surface of the AgNPs. The second method of attachment is through bio-conjugation, namely, the formation of chemical bonds between the biological molecule and stabilizer molecules attached to the surface of the silver nanoparticles [26]. However, bioconjugation reactions, in most cases, have not been optimized, such that attachment of the desired molecule to the nanoparticle surface is guaranteed. Hence, optimization is needed in future studies on this noncovalent interaction of plant extracts with nanoparticles.

The hybrid molecules were synthesized separately and characterized by FTIR to identify functional groups that confirm the conjugation process. Plant extracts, when conjugated with silver nanoparticles, have revealed the presence of C-H and carbonyl (C=O) groups. However, -OH carbonyl and C≡C group were not observed in the individual solutions before conjugation. These peaks are due to the organic compounds which are present in the extract and responsible for silver ions reduction and stabilization of resultant nanoparticles [27]. However, the functionalization of biomolecules with silver nanoparticles are known to have a primary amine group, a carbonyl group, hydroxyl groups, and other stabilizing functional groups, as shown by FTIR spectroscopic technique [28]. The temperature-responsiveness, stability and cytotoxicity of these conjugates should be evaluated since studies indicated that it is of crucial importance, and there is a strong dependence of cytotoxicity and geno-toxicity on Ag-NPs functionalities, composition, wettability, and thickness [29,30].

In hospitals, infection is the most common complication and cause of death in patients. Therefore, the antibacterial effects of silver have been incorporated into various medical applications. Plastic catheters coated with silver nanoparticles prevent biofilm formation from *E. coli*, *Enterococcus Staphylococcus aureus, Candida albicans, Staphylococci*, and *Pseudomonas aeruginosa* and also show significant in vitro antimicrobial activity [17]. In our study, well diffusion assay results clearly indicated that chemically synthesized silver nanoparticles, when conjugated with *P. grandiflora* tuber extracts, have good antibacterial activity against *E. coli*, *K. pneumonia*, and *S. aureus*. Similarly, in a recent report, these nanoparticles have been synthesized via irradiation using an aqueous mixture of *Ficus carica* leaf extract [20] *Cymbopogan citratus* (DC) stapf (commonly known as lemon grass), a native aromatic herb from India, and also cultivated in other tropical and subtropical countries, showed strong antibacterial effect against *P. aeruginosa*, *P. mirabilis*, *E. coli*, *Shigella flexneri*, *S. Sonei* and *K. pneumonia* [31].

The green rapid syntheses of spherically shaped silver nanoparticles with dimensions of 50–100 nm were observed using *Alternanthera dentate* aqueous extract [32]. Comparison to silver nanoparticles synthesized in this study shows that they were much smaller in size than those reported by Kumar and colleagues. The silver nanoparticles reported by Kumar and colleagues exhibit antibacterial activity against *P. aeruginosa*, *E. coli*, *K. pneumonia*, and *E. faecal* [33]. In this scenario, the highest zone of growth inhibition of 24 mm was observed with biologically synthesized silver nanoparticles conjugated with acetone extract against MRSA. Nevertheless, chemically synthesized silver nanoparticles showed the highest zone of growth inhibition with a diameter of 31 mm against *S. aureus* when conjugated with both acetone and methanol extracts. Hence, acetone extracts can be considered as the best extract for conjugation since they exhibited higher antibacterial activity.

The MIC results revealed that all extracts were active at a very low concentration, lower than 1 mg/mL of plant extracts [30]. The study conducted by Nabikhan et al. [24] has revealed that silver nanoparticles conjugated with *Sesuvium portulacastrum* active compounds as stabilizers showed high antimicrobial activities against *S. typhi*, *E. coli, S. aureus*, and *B. subtilis* microorganisms. In this study, the lowest MIC was revealed when biologically synthesized silver nanoparticles are conjugated with acetone and water extract against methicillin-resistant *Staphylococcus aureus* with the MIC value of 0.0063 mg/mL. The overall synergistic effect was (23.3%), whereas (30%) were additive and (46%) were antagonism. However, the most synergistic effect was observed with *P. grandiflora* acetone and water extract when conjugated with chemically synthesized nanoparticles against *E. coli* and *K. pneumoniae*. 

## 5. Conclusions

The aim of this study was to develop new strategies that could be used for the management of infectious diseases. Synthesis and characterization of *P. grandiflora* acetone and water extracts conjugated with silver nanoparticles were successfully carried out in this study. The in-vitro antibacterial activity of the mixtures showed a significant increase against multi-drug resistant pathogens, such as methicillin-resistant *Staphylococcus aureus*, beta-lactamase-producing *Klebsiella pneumonia*, and beta-lactamase-producing *Escherichia coli*. Biologically synthesized silver nanoparticles showed to be less effective compared to chemically synthesized silver nanoparticles. Well diffusion assay results clearly indicated that chemically synthesized silver nanoparticles when conjugated with *P. grandiflora* tubers extracts had good bacterial activity against *E. coli, K. pneumoniae,* and *S. aureus* when compared to biologically synthesized silver nanoparticles conjugates. Therefore, biosynthesized conjugates could be utilized as antimicrobial agents for effective disease management due to the synergistic antibacterial activity that was observed. The results of this study could find application in the preparation of a variety of antimicrobial products that can be used in medicine or other biotechnological applications.

## Figures and Tables

**Table 1:** Minimum inhibitory concentration (MIC) of biologically and chemically synthesized silver nanoparticles, as well as their conjugates with P. grandiflora extracts.

Antibacterial Agents	*E. coli* 25922	*E. coli* 35218	*K. pneumonia*	MSSA	MRSA
	Biologically synthesized silver nanoparticles				
SB	0.4	0.1	0.1	0.1	0.8
SA	0.05	0.1	0.1	0.1	0.0063
SM	0.8	0.2	0.2	0.8	0.0063
SW	0.8	0.8	0.8	0.1	0.2
	Chemically synthesized silver nanoparticles				
SC	0.0063	0.8	0.8	0.05	0.2
SA	0.0063	0.0125	0.0125	0.05	0.8
SM	0.4	0.0063	0.0063	0.05	0.8
SW	0.4	0.2	0.2	0.1	0.8

Key: SB (Biologically synthesized silver nanoparticles), SA (Silver nanoparticles and acetone extract), SM (Silver nanoparticles and methanol extract), SW (Silver nanoparticles and water extract) and SC (Chemically synthesized silver nanoparticles).

**Table 2:** Minimum bactericidal concentration (MBC) of chemically synthesized silver nanoparticles, as well as their conjugates.

Antibacterial Agents	*E. coli* 25922	*E. coli* 35218	*K. pneumonia*	MSSA	MRSA
SC	0.4	0	0	0	0
SA	0.05	0	0	0.0063	0
SM	0	0.0063	0	0.0063	0
SW	0	0	0	0.2	0

Key: SC (Chemically synthesized silver nanoparticle), SA (Silver nanoparticles and acetone extract), SM (Silver nanoparticles and methanol extracts), SW (Silver nanoparticles and water extracts), and 0 (Bacterial growth observed indicating non-activity in terms of the bactericidal capability).

**Table 3:** Effect of conjugating *P. grandiflora* tuber extracts with silver nanoparticles.

Conjugates	*E. coli* 25922	*E. coli* 35218	*K. pneumonia*	MSSA	MRSA
SA_B_	0.25 (S)	1.125 (A)	1.125(A)	1.125(A)	0.023625(S)
SM_B_	18 (N)	3(A)	2.5(A)	9.290323(N)	0.070875(N)
SW_B_	18(N)	10(N)	134.9841(N)	16.87302(N)	0.5(S)
SA_C_	1.01575(A)	0.03125(S)	0.03125(S)	1.0625(A)	6(N)
SM_C_	71.49206(N)	0.01575(S)	0.023625	1.080645(A)	12(N)
SW_C_	71.49206(N)	0.75(A)	31.99603(N)	17.87302(N)	5(N)

Key: _B_ (Biological synthesis), _C_ (Chemical synthesis), SA (Silver nanoparticles and acetone extract), SM (Silver nanoparticles and methanol extracts), SW (Silver nanoparticles and water extracts). A (additive), N (antagonism) and S (synergy).

## Data Availability

Not applicable.
